# Severe Hyperkalemia With a Normal Electrocardiogram: Pseudohyperkalemia in Extreme Leukocytosis

**DOI:** 10.7759/cureus.106856

**Published:** 2026-04-11

**Authors:** Yasir Kammawal, Nasir Khan Kamawal

**Affiliations:** 1 Internal Medicine, St. Mary's Hospital, Isle of Wight NHS Trust, Newport, GBR; 2 Department of Pediatrics, Nangarhar University Teaching Hospital, Jalalabad, AFG

**Keywords:** electrolyte disorder, hematologic malignancy, hyperkalemia, laboratory artifact, leukocytosis, point of care testing, potassium discrepancy, preanalytical error, pseudohyperkalemia, whole blood potassium

## Abstract

Pseudohyperkalemia is an important laboratory artifact that may lead to unnecessary and potentially harmful treatment if not recognized promptly. We report a case of apparent severe hyperkalemia in a patient with extreme leukocytosis, in whom repeated serum potassium measurements remained markedly elevated despite the absence of clinical symptoms or electrocardiographic abnormalities. Given the discordance between biochemical findings and clinical presentation, further evaluation was undertaken using point-of-care whole blood analysis, which demonstrated a normal potassium level. The discrepancy was consistent with pseudohyperkalemia due to in vitro leukocyte lysis during sample processing. Recognition of this phenomenon prevented inappropriate potassium-lowering therapy. This case highlights the importance of correlating laboratory results with the clinical picture and emphasizes the value of alternative testing modalities in resolving diagnostic uncertainty.

## Introduction

Hyperkalemia is a potentially life-threatening electrolyte abnormality associated with an increased risk of cardiac arrhythmias and requires prompt recognition and management. Its clinical significance depends on both the absolute potassium level and the rate of rise, with electrocardiographic (ECG) changes providing an important indicator of physiological effect [1].

However, not all elevated potassium measurements represent true hyperkalemia. Pseudohyperkalemia refers to an artificial increase in measured potassium concentration caused by potassium release from blood cells during or after sample collection, rather than reflecting the patient’s in vivo physiological state [1,2]. This phenomenon has been described in association with hemolysis and marked leukocytosis [1-3].

In cases of extreme leukocytosis, particularly in hematologic disorders, leukocytes are structurally fragile and susceptible to mechanical disruption. This may result in intracellular potassium leakage during venipuncture, clot formation, centrifugation, or specimen transport, leading to falsely elevated serum potassium levels [1,3]. Pre-analytical factors, including vacuum collection systems and delayed sample processing, may further accentuate this effect [4].

Failure to recognize pseudohyperkalemia may lead to unnecessary and potentially harmful treatment, including insulin-glucose therapy, beta-agonists, or calcium administration, which can precipitate true hypokalemia and related complications [4]. Distinguishing true hyperkalemia from pseudohyperkalemia, therefore, requires careful correlation of laboratory findings with the clinical picture, along with consideration of alternative testing methods such as whole blood analysis [2].

We present a case of severe apparent hyperkalemia in the setting of extreme leukocytosis in which the absence of clinical and electrocardiographic abnormalities prompted further evaluation and led to the diagnosis of pseudohyperkalemia, thereby avoiding inappropriate treatment.

## Case presentation

An elderly male presented with a short history of generalized fatigue and reduced oral intake. He denied chest pain, palpitations, syncope, or muscle weakness. His past medical history included hypertension. Recent blood tests had demonstrated marked leukocytosis, for which outpatient hematology evaluation was ongoing. He had no known chronic kidney disease and was not taking any medications known to increase serum potassium levels.

On admission, he was hemodynamically stable with normal vital signs. Physical examination was otherwise unremarkable.

Initial laboratory investigations demonstrated a serum potassium level of 7.1 mmol/L, with preserved renal function (creatinine 92 µmol/L, urea 6.9 mmol/L). Given the severity of the hyperkalemia, repeat testing was performed and again showed a markedly elevated potassium level of 7.4 mmol/L, while renal function remained stable (creatinine 95 µmol/L, urea 7.1 mmol/L).

**Figure 1:**
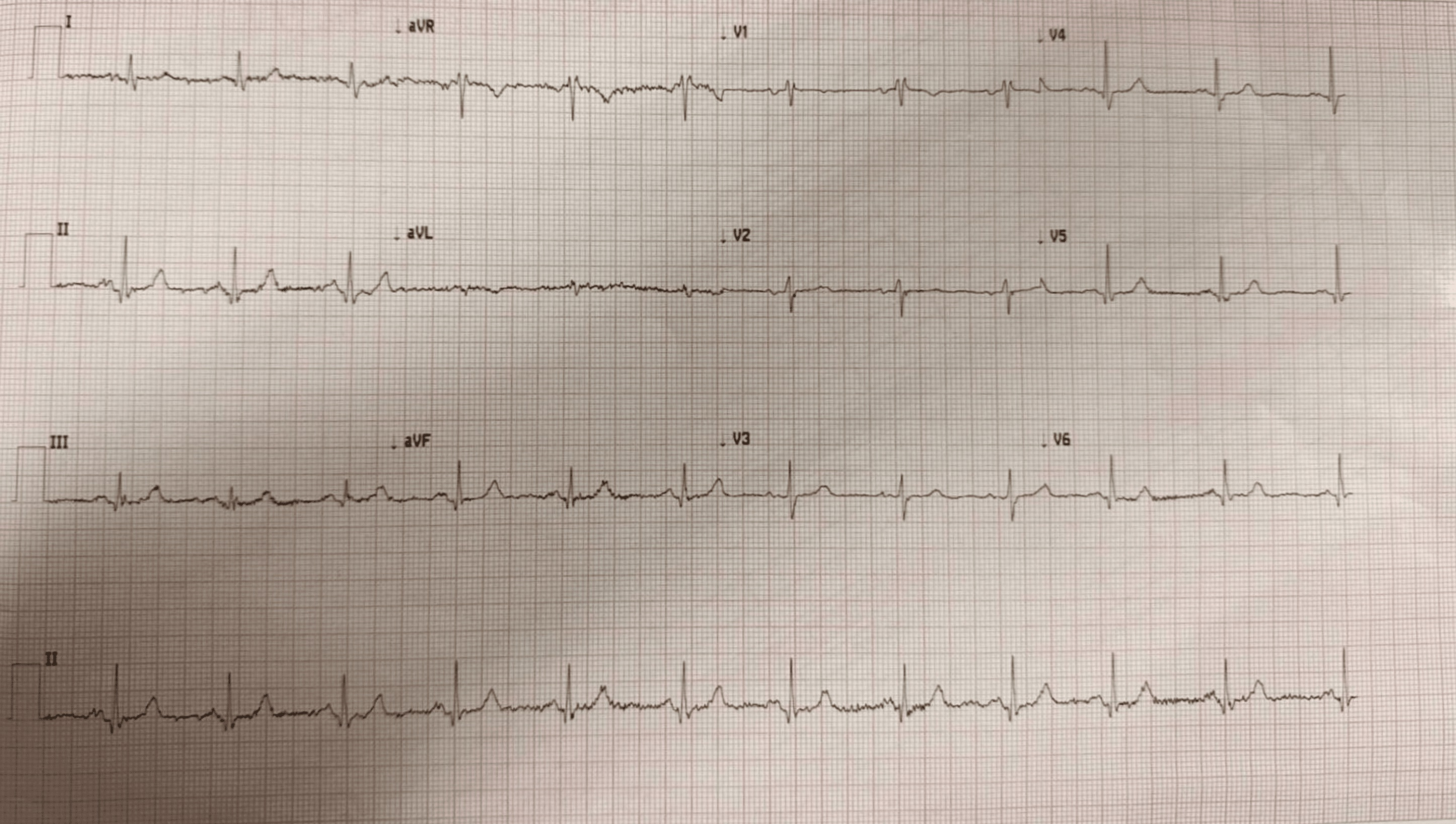
Electrocardiogram demonstrating absence of hyperkalemic changes Twelve-lead electrocardiogram demonstrating normal sinus rhythm without classical features of hyperkalemia, including absence of peaked T waves, QRS widening, or conduction abnormalities.

Despite these biochemical findings, the patient remained clinically asymptomatic. Electrocardiography demonstrated normal sinus rhythm without features of hyperkalemia, including absence of peaked T waves, QRS widening, or conduction abnormalities (Figure 1).

**Figure 2:**
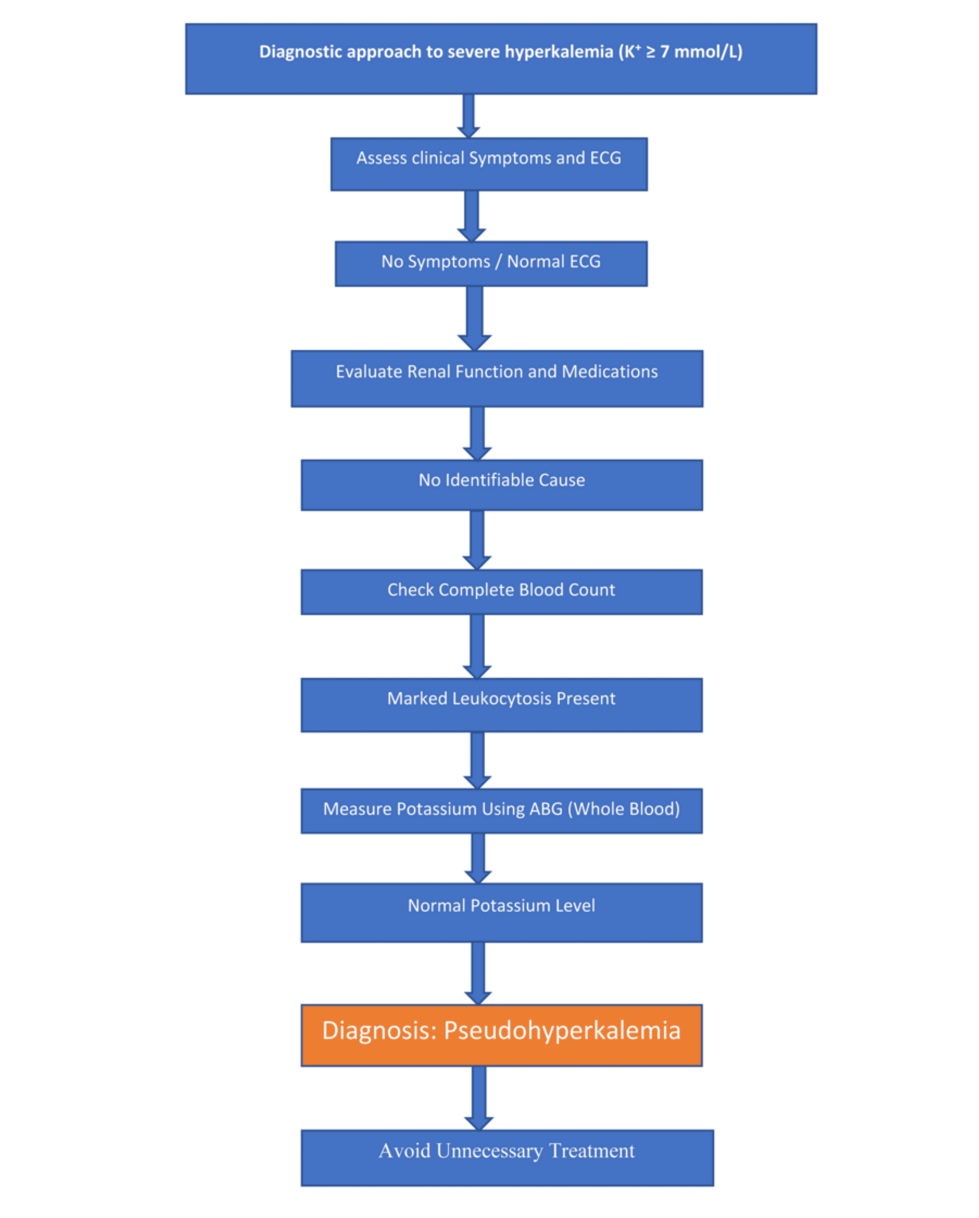
Diagnostic approach to suspected pseudohyperkalemia Stepwise diagnostic approach demonstrating evaluation of severe hyperkalemia with discordant clinical findings, including exclusion of true causes, identification of leukocytosis, and confirmation using whole blood potassium measurement. ABG: arterial blood gas

The marked discordance between the biochemical severity of hyperkalemia and the absence of clinical or electrocardiographic abnormalities prompted further evaluation using a structured diagnostic approach (Figure 2). Medication review did not identify any contributing agents, and renal function remained within normal limits. A diagnostic assessment was therefore undertaken. True hyperkalemia was considered unlikely given preserved renal function and absence of potassium-raising medications. The lack of symptoms and a normal electrocardiogram further reduced the likelihood of clinically significant hyperkalemia. In the context of marked leukocytosis, pseudohyperkalemia due to in vitro cell lysis was considered highly likely.

Subsequent laboratory analysis revealed marked leukocytosis, with the white blood cell count rising from 168 × 10⁹/L to 178 × 10⁹/L. Hemoglobin was mildly reduced but stable (11.1 g/dL initially and 11.0 g/dL on repeat testing), while platelet count remained within the normal range (318 × 10⁹/L initially and 312 × 10⁹/L on repeat testing).

To further assess the elevated potassium levels, a point-of-care arterial blood gas (ABG) sample was obtained using heparinized whole blood. This demonstrated a potassium level of 4.3 mmol/L with normal acid-base status (arterial pH 7.38).

Comparison of serum potassium with whole blood potassium values demonstrated a significant discrepancy, further supporting pseudohyperkalemia. There was no laboratory evidence of hemolysis in the analyzed samples, making red cell lysis an unlikely cause of the elevated potassium levels. Key laboratory findings are summarized in Table 1.

**Table 1:** Laboratory Findings ABG: arterial blood gas; POC: point of care

Parameter	Initial	Repeat	ABG (POC)	Reference Range
Potassium (mmol/L)	7.1	7.4	4.3	3.5–5.0
Creatinine (µmol/L)	92	95	—	60–110
Urea (mmol/L)	6.9	7.1	—	2.5–7.8
White blood cell count (×10⁹/L)	168	178	—	4–11
Hemoglobin (g/dL)	11.1	11	—	13–17
Platelets (×10⁹/L)	318	312	—	150–400
Arterial pH	—	—	7.38	7.35–7.45

The marked discrepancy between serum potassium (7.1-7.4 mmol/L) and whole blood potassium (4.3 mmol/L) was a key diagnostic finding, supporting pseudohyperkalemia rather than true hyperkalemia. This difference is clinically significant, as whole blood potassium measurement minimizes in vitro cellular breakdown. The extreme leukocytosis (168-178 × 10⁹/L) likely contributed to potassium release during sample processing, explaining the falsely elevated serum values.

Clinical course

The final diagnosis was pseudohyperkalemia secondary to extreme leukocytosis, most likely due to in vitro leukocyte lysis during sample processing. There was no laboratory evidence of hemolysis in the analyzed samples, making red cell lysis an unlikely contributor. Potassium-lowering therapy was withheld. The patient remained clinically stable throughout admission, and subsequent monitoring using whole blood potassium measurements continued to demonstrate values within the normal range. The patient was referred for ongoing hematology evaluation to determine the underlying cause of leukocytosis.

## Discussion

Pseudohyperkalemia is a recognized laboratory phenomenon in which measured potassium levels are falsely elevated due to in vitro release of intracellular potassium, rather than reflecting true hyperkalemia in vivo. Although most commonly associated with hemolysis, it is also described in settings of marked leukocytosis [1-3].

In patients with significant leukocytosis, particularly in hematologic disorders, leukocytes may be structurally fragile and highly susceptible to mechanical stress. During venipuncture, clot formation, centrifugation, or specimen transport, these fragile cells may undergo lysis, leading to release of intracellular potassium into the serum sample. This results in spuriously elevated potassium levels that do not correspond to the patient’s physiological state [1,3].

Recent literature has further emphasized that pre-analytical factors play a critical role in this process. Mechanical stress during sample handling, particularly pneumatic tube transport systems, has been associated with an increased risk of pseudohyperkalemia in patients with extreme leukocytosis, with the effect becoming more pronounced at very high white blood cell counts [5].

The distinction between true hyperkalemia and pseudohyperkalemia is clinically important. True hyperkalemia is associated with potentially life-threatening cardiac arrhythmias and typically manifests with electrocardiographic changes such as peaked T waves, QRS widening, and conduction abnormalities. However, the absence of such findings, particularly in the presence of markedly elevated potassium levels, should prompt reconsideration of the diagnosis. In the present case, despite potassium levels exceeding 7 mmol/L, the patient remained asymptomatic with a normal electrocardiogram (Figure 1), emphasizing the importance of correlating laboratory values with the clinical picture.

A key diagnostic clue in this case was the presence of extreme leukocytosis. Previous studies have demonstrated that pseudohyperkalemia is more likely when white blood cell counts exceed 100 × 10⁹/L, with the risk increasing at higher levels [2]. More recent evidence suggests that this risk may be further increased at very high counts, particularly above 200 × 10⁹/L, where leukocyte fragility and susceptibility to mechanical disruption are more pronounced [5,6].

Point-of-care testing using whole blood analysis, such as ABG sampling, plays an important role in resolving this diagnostic uncertainty. Whole blood samples are processed rapidly, avoiding centrifugation and clotting steps that contribute to cellular lysis. In this case, the ABG potassium level was within normal limits, supporting the conclusion that the elevated serum potassium represented a laboratory artifact rather than true hyperkalemia. This finding is consistent with reports demonstrating that whole blood potassium measurement more accurately reflects in vivo potassium levels in suspected pseudohyperkalemia [6].

Failure to recognize pseudohyperkalemia may lead to inappropriate and potentially harmful interventions. Standard treatments for hyperkalemia, including insulin-glucose therapy, beta-agonists, and calcium administration, are associated with risks and may precipitate iatrogenic hypokalemia or cardiac complications when given unnecessarily [4]. Case-based literature further highlights that unnecessary treatment in such scenarios may expose patients to avoidable adverse effects, reinforcing the importance of diagnostic confirmation prior to intervention [7].

This case underscores a fundamental clinical principle: laboratory results must always be interpreted alongside the patient’s presentation. In this scenario, the marked discordance between biochemical findings and clinical stability served as the key diagnostic clue. A structured approach incorporating repeat testing, assessment of possible contributing factors, and use of alternative measurement techniques enabled accurate diagnosis and safe management.

## Conclusions

Pseudohyperkalemia should be considered in patients with markedly elevated serum potassium levels that are inconsistent with clinical findings, particularly in the presence of extreme leukocytosis. Failure to recognize this phenomenon may lead to unnecessary and potentially harmful treatment.

This case highlights the importance of correlating laboratory findings with the clinical assessment and underscores the role of alternative testing methods, such as whole blood potassium measurement, in resolving diagnostic uncertainty. A structured diagnostic approach can help clinicians distinguish true hyperkalemia from laboratory artifacts, thereby preventing inappropriate interventions and improving patient safety.

This observation is based on a single case and should be interpreted with caution; however, it highlights an important diagnostic consideration in clinical practice.

Ultimately, this case reinforces a fundamental principle of clinical medicine: treat the patient, not the number.

## References

[1] Meng QH, Wagar EA (2015). Pseudohyperkalemia: a new twist on an old phenomenon. Crit Rev Clin Lab Sci.

[2] Rifkin SI (2011). Pseudohyperkalemia in patients with chronic lymphocytic leukemia. Int J Nephrol.

[3] Sevastos N, Theodossiades G, Archimandritis AJ (2008). Pseudohyperkalemia in serum: a new insight into an old phenomenon. Clin Med Res.

[4] Asirvatham JR, Moses V, Bjornson L (2013). Errors in potassium measurement: a laboratory perspective for the clinician. N Am J Med Sci.

[5] Tseng YC, Lin PB, Hsieh S (2024). Pneumatic tube transport-induced pseudohyperkalemia in patients with extreme leukocytosis: a retrospective study from a single medical center. Int J Hematol.

[6] Ghumman GM, Baqi A, Adil AN, Khatri V (2022). Role of point-of-care arterial blood potassium in diagnosing pseudohyperkalemia. Proc (Bayl Univ Med Cent).

[7] Arani N, Wechsler AH (2023). Hyperkalemia in the setting of severe leukocytosis: should you treat?. Am J Emerg Med.

